# How many species in the United States warrant consideration for Endangered Species Act protection?

**DOI:** 10.7717/peerj.20692

**Published:** 2026-01-27

**Authors:** Noah Greenwald, Krista Kemppinen, Will Harlan, Jeff Miller

**Affiliations:** 1 Center for Biological Diversity, Portland, OR, United States of America; 2 Center for Biological Diversity, Tucson, AZ, United States of America; 3 Center for Biological Diversity, Asheville, NC, United States of America; 4 Center for Biological Diversity, Ashland, OR, United States of America

**Keywords:** Endangered species, Conservation policy, Regulatory protection, Biodiversity threats

## Abstract

The U.S. Endangered Species Act (“ESA”) currently protects 1,682 species as endangered or threatened. According to the independent scientific organization NatureServe, however, there are more than 10,000 imperiled species in the United States that may need protection. One barrier to protecting recognized imperiled species is a lack of threats information. To address this problem, we reviewed all species recognized as critically imperiled (G1) or imperiled (G2) by NatureServe to identify those facing documented threats. In total, we identified 2,204 species where there is sufficient threat information to indicate ESA protection may be warranted. This analysis indicates that more than double the species currently listed under the ESA may need protection to avoid extinction. The U.S. Fish and Wildlife Service (“Service”) has on average listed just 32 species per year since the law was passed in 1973. At this rate, most species currently recognized as imperiled and facing threats will not receive consideration for protection within any meaningful timeframe.

## Introduction

The Endangered Species Act (“ESA”) is widely regarded as one of the most powerful laws for protecting wildlife ever enacted (Bean & Rowland, 1997). The ESA provides strong protections to species, including a blanket prohibition against “take”, which includes any action that causes the death, injury, or harassment of protected species, or the destruction of their habitat, and an affirmative duty for all federal agencies to avoid jeopardizing the continued existence of listed species or adversely modifying their critical habitat. These strong protections, however, only apply once a species has been “listed” as a threatened or endangered species, and thus listing is in many ways the keystone of the ESA.

Despite its importance, the US Fish and Wildlife Service (“Service”) has been chronically slow to list species, frequently taking a decade or more to complete a process that under the statute is required to take no more than two years (Greenwald, Suckling & Taylor, 2005; Puckett, Kesler & Greenwald, 2016; Eberhard, Wilcove & Dobson, 2022). In part because of this slow pace, the ESA’s protections have been applied to only a fraction of those species at risk of extinction. Wilcove & Master (2005), for example, estimated that the number of species threatened with extinction is “at least ten times greater than the number protected” under the ESA. Similarly, Harris et al. (2012) found that 40% of birds on the International Union for Conservation of Nature (IUCN) Red List are not listed under the ESA and that most other groups are underrepresented by >80%.

One barrier to protecting more species under the ESA is a lack of information on threats to their survival. The independent scientific organization NatureServe has recognized more than 10,000 species as either critically imperiled (G1) or imperiled (G2). The available information for many if not most of these species, however, is highly limited. Although there can be little question these species are rare, the ESA requires documentation of threats before protection is provided.

The Service places additional scrutiny on listing decisions above and beyond the criteria utilized by organizations like NatureServe or IUCN. According to the Service, the “mere identification of any threat(s) does not necessarily mean that the species meets the statutory definition of an ‘endangered species’ or a ‘threatened species”’ (See, for example, USFWS, 2024). Beyond identification of threats, the Service analyzes the “expected response by the species” and the effects of the threats, considering ameliorating factors, on the species viability. There is simply not sufficient information to conduct such an analysis for many of the species NatureServe or other organizations identify as imperiled.

To identify species that may meet the Service’s heightened scrutiny, and ensure species facing threat are considered for protection, we developed a method for rapidly assessing NatureServe species accounts. We first deployed this methodology in 2010 to file a petition to list 404 aquatic and wetland species from the southeastern US under the ESA (CBD, 2010). In response, the Service issued a positive initial finding for 374 of the 404 species. The Service considered another 18 species to already be candidates for listing and thus treated the petition as redundant, and another two were determined to be extinct, meaning that just 10 of the 404 species were denied consideration for ESA listing because of insufficient or contrary information.

We herein present the method we used to identify these 404 species and apply that methodology to all species identified as critically imperiled (G1) or imperiled (G2) by NatureServe. In so doing, we present a condensed list of species with sufficient information on threats to their survival such that they warrant consideration for ESA protection and should be priorities for further study by the US Fish and Wildlife Service to avoid extinction. We also present information on the taxonomy, geography and threats to these species.

## Methods

The ESA allows any person to petition to list a species as endangered or threatened and requires the Service to make a series of required findings in response to these petitions. The first of these findings, often referred to as a “90-day finding” for the time it is supposed to take, determines whether the petition presents sufficient information to warrant further consideration. The standard the Service uses is whether a “reasonable person” reviewing the petition would find it presented sufficient information to indicate the species meets the definition of an endangered (at risk of extinction in all or a significant portion of range) or threatened (at risk of becoming endangered in all or a significant portion of range) species. If this initial finding is positive, the Service then conducts a status review to determine if listing is warranted, in which case protection is proposed. Our aim in this study is to identify those species that would meet this initial 90-day hurdle and warrant a status review with the petition for the 404 Southeast species showing our methodology effective.

We began our analysis by extracting from the NatureServe database all G1 and G2 species that had some text in the “Threat Comments” field (NatureServe, 2018). This produced 4,518 results. We then reviewed each of these species to determine if there was sufficient information on threats to indicate risk to the species such that it would meet the 90-day hurdle, in which case we would categorize the species as “may warrant” for protection. In 2024, we obtained updated data from NatureServe including species newly recognized as G1 or G2 or species no longer recognized as such and added this information to our analysis (NatureServe, 2024). Species that were already listed or proposed for listing were excluded from analysis. We also excluded those species that had in the last 10 years, received a negative 90-day or 12-month finding, or been the subject of a listing rule withdrawal or delisting decision.

To make the “may warrant” determinations, we relied primarily on NatureServe’s stated reasons for the species’ global status (“G_RANK_REASONS”) and comments provided on threats (“G_THREAT_COM”). We categorized species as “may warrant” if one or more external threats were identified (Table 1). We excluded species where NatureServe described threats as highly uncertain or unknown, localized to a small portion of a species’ range, or minimal (Table 2).

In addition to evaluating threats, we also considered information on abundance, numbers of populations and population trend available in several other NatureServe fields. For most species tracked by NatureServe, the information available in these fields tends to be course and sparse, but to the extent it showed species to have low abundance or sharply declining trends, we factored that into our may warrant classifications. We also considered rankings by the International Union for the Conservation of Nature and the American Fisheries Society where available with endangered or similar classifications by these organizations weighing in favor of a may warrant classification.

For each species found to “may warrant” protection, we classified threats according to the five factors identified in the ESA for determining whether species warrant listing, which include (A) present or threatened destruction, modification or curtailment of habitat or range; (B) over-utilization; (C) disease or predation; (D) inadequacy of existing regulatory mechanisms, or (E) other natural or manmade factors affecting a species’ continued existence, which includes climate change, invasive species, altered disturbance regime, small population size and others (16 U.S.C. §1533(a)(1)).

**Table 1:** Examples of species categorized as may warrant due to having one or more external threats.

Common name	Scientific name	Threat comments	Reasons for global status
Fly Ranch Pyrg	*Pyrgulopsis bruesi*	The spring area where this snail occurs has been modified by surface water diversion, groundwater mining, dredging, and recreational activity (Hershler & Sada, 2000).	Endemic to one large thermal spring area in northwest Nevada.
Rio Grande Darter	*Etheostoma grahami*	Threats include pollution, reduction of water flow, and, in some cases, elimination of water flow. Jelks et al. (2008) categorized this species as Threatened, based on present or threatened destruction, modification, or reduction of habitat or range.	Small range in streams in southern Texas and northeastern Mexico; low overall abundance; vulnerable to local reductions in water flow.

**Table 2:** Examples of species not categorized as “may warrant” due to being clearly described by NatureServe as having an overall low degree of threat or highly uncertain/unknown threats.

Common name	Scientific name	Threat comments	Reasons for global status
Diablo Range Pyrg	*Pyrgulopsis diablensis*	It is unclear whether there are any threats affecting this species. Hershler (1995) records disturbance of habitat by pastoral and recreational activities but the extent to which the population is affected is not known.	Although only known from a single creek in California, the species was only recently described and threat and trend information are lacking.
Strecker’s Pocket Gopher	*Geomys streckeri*	This pocket gopher is not known to be threatened, but its small distribution makes it vulnerable to unfavorable habitat alteration	Very small range in Texas; vulnerable to unfavorable habitat alteration; regarded by some authorities as a subspecies of Geomys personatus.
Potato-chip Lichen	*Omphalora arizonica*	The reason for its variable abundance at different locations is unknown. Threats appear to be few. Threats include air pollution that is likely to be a problem only in a few parts of its range. Mechanical disturbance such as rock climbing may impose a threat in locations such as the Sandia Mountains of New Mexico but is unlikely to be a significant concern.	Omphalora arizonica is a rare lichen although it has a relatively wide range and imprecise habitat requirements. Egan (1972) suggests that it was once much more widely distributed in the southwestern United States but is now surviving well in only a few areas and in some areas appears to be almost extinct.
Brian Head Mountainsnail	*Oreohelix parawanensis*	There appear to be few immediate threats excepting the fact that the species occurs as a single, localized population and is susceptible to catastrophic events. Collecting or survey disturbance could potentially impact the species if specimens were overcollected or trampled. Nearby ski resorts do not threaten the snail but if resort operations were to expand, the entire population could be destroyed (Oliver & Bosworth III, 2002). Other potential threats include hikers and mountain bikers, who utilize the area, and domestic sheep, which have been observed in large numbers not far away. Because it exists above the tree line, timber harvest is not a threat (Oliver & Bosworth III, 2002).	Known, since ca. 1935, only from the type locality, Brian Head (Peak), Iron County, Utah, where in 1998 the first living examples were discovered. Only an estimated 2.3 ha may actually be inhabited. Surveys elsewhere in the region have not revealed any other populations. Although restricted to a single site, it is at high elevation and away from human disturbance. Potential threats are minimal.
Salt Creek Pupfish	*Cyprinodon salinus*	Potential threats include introduction of non-native species, localized catastrophic events, and excessive pumping of the aquifer that feeds the habitat (Moyle, 2002).	Comprises only two populations, both in Death Valley National Park, California; population size sometimes very large but highly variable; no significant threats, but overall habitat is very small and vulnerable to events that could quickly reduce or eliminate the populations.

## Results

**Figure 1:**
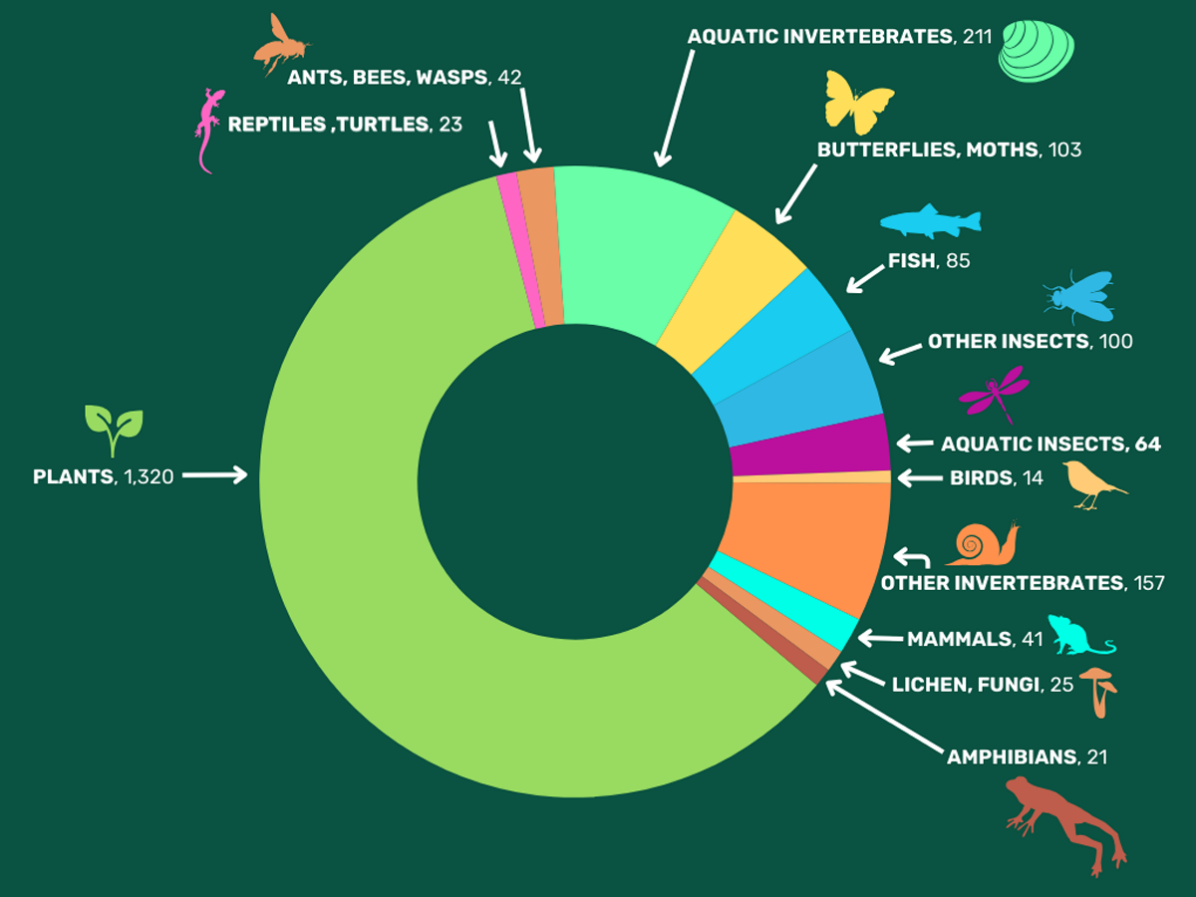
Taxonomic classifications of 2,206 imperiled species that may warrant protection under the Endangered Species Act.

In total, we identified 2,204 species that may warrant protection as endangered or threatened species under the ESA. Of these, a majority are plants (1,320 species, 60 percent), followed by insects (309, 15 percent), including 103 butterflies and moths and 42 ants, bees and wasps, terrestrial snails (115, five percent), freshwater snails (90, four percent), fish (85, four percent), lichen and fungi (25, one percent), reptiles and turtles (23, 1 percent), amphibians (21, one percent), and birds (14, 0.6 percent) among others (Fig. 1). Freshwater species are well represented, including the 85 fish, 90 freshwater snails, 82 crayfish, 64 aquatic insects and 26 freshwater mussels.

Habitat destruction was identified as a threat to most of the 2,204 species (2,023, 92 percent), followed by invasive species (731, 33 percent), small population size (576, 26 percent), climate change (387, 18 percent), altered disturbance regime (271, 12 percent), disease and predation (185, eight percent), over-utilization (153, seven percent), and inadequacy of existing regulations (84, four percent).

The western US had the most species that may warrant protection (1,105 species) with the majority of those found in California (688 species), followed by the Southeast (650 species), Southwest (323 species), Midwest (88 species) and Northeast (84 species). Despite already having the most listed endangered and threatened species of any state, Hawaii had 169 additional species that need consideration for protection.

## Discussion

Prior to 1996, the Service maintained lists of “category 2 candidate” species where “listing was possibly appropriate, but for which sufficient data on biological vulnerability and threat were not currently available to support proposed rules”, including separate lists for plants and animals which combined included nearly 3,500 species (USFWS, 1996). The Service eliminated these lists in 1996, instead only maintaining a list of species already found to warrant protection, but for which the agency purported to lack sufficient resources to provide protection, and which currently includes just 16 species, all found outside of the United States (USFWS, 2025).

The Service and the public at large thus lack a current list of species that merit consideration for ESA listing. Without such a list, the Service is unable to request resources from Congress to carry out listing determinations proportionate to need, and public and private interests are left unaware of what species may be subject to future regulation. As such, the 2,204 species identified in this study fill an important gap, identifying a suite of species that should be the focus of conservation, study and ultimately consideration for listing as endangered or threatened species under the ESA.

The 2,010 petition for 404 southeastern aquatic and wetland species had just this effect, leading to extensive monitoring and research of southeast species (*e.g.*, Burington, Morse & McArthur, 2012; Rhoden, Taylor & Wagner, 2016; McCall, 2017; Grubbs, 2021). Based on this research, the Center has withdrawn 139 species from consideration based on taxonomic revisions, discovery of more populations or reduced threat. To date, the petition has resulted in 51 species receiving ESA listing (CBD, unpublished data, 2025). Another 159 species are still waiting for decisions, 15 years later, and 71 have been denied protection. The Center challenged six of these denials in federal court, leading to pending new decisions for four species, proposed listing of one (eastern hellbender), and one that is still before the courts. Undoubtedly, some proportion of the 2,204 species identified in this study will similarly be determined to not warrant ESA protection. From our perspective, the additional research and monitoring to demonstrate species are at least nominally secure is effort well-expended.

The inclusion of hundreds of freshwater animals in our list of may warrant species is consistent with studies finding accelerated extinction rates in North American freshwater species (Master, 1990; Ricciardi & Rasmussen, 1999) and likely contributes to the large number of imperiled species in the southeastern U.S., which is a hotspot for aquatic biodiversity (Folkerts, 1997). The large number of imperiled species in California in contrast, reflects the abundant floral diversity of the state with plants comprising 537 of the 680 imperiled species needing consideration for protection (Myers et al., 1999).

Comprising just 609 of the 2,204 species, invertebrate animals are clearly underrepresented in our list of species needing consideration for protection, reflecting the fact that many groups are poorly studied including many undescribed species (Wilcove & Master, 2005). Likewise, the inclusion of just 25 fungi and lichen species reflects the little study these groups have received.

Our finding that habitat destruction and invasive species, in that order, were most often identified as threats to imperiled species is consistent with previous studies, most notably Wilcove et al. (1998), who also looked at threats to NatureServe identified imperiled species. When this seminal study was conducted, climate change was not listed as a threat to any species, but the authors observed that “it is almost certain to become one in the foreseeable future due to increasing concentrations of greenhouse gases from fossil-fuel use, land-use changes, and agriculture” (Wilcove et al., 1998). Climate change was identified as a threat to 387 (18 percent) of the 2,204 species. With studies reporting that as many as one-third of all species globally may be at risk of extinction due to climate change (see Urban, 2024), we suspect this is an underestimate and that many more species in the United States are at risk of climate change driven extinction.

NatureServe does not include data or analysis of regulatory protections afforded to species and thus our finding that a mere 84 species are threatened by a lack of regulatory protections does not represent reality. In the case of these 84 species, review authors specifically noted lack of protection in the G-Rank Reasons or the Threats Comments fields, which is not a uniform practice.

Scott et al. (2005) recognized that many imperiled species are conservation reliant, meaning their survival hinges on active management. We identified altered disturbance regime as a threat to 271 (12 percent) of the 2,204 species, which most often consisted of either a lack of or increase in wildfires, in either case necessitating corrective action. Another 731 (33 percent) of the species were identified as facing potential threats from invasive species likewise necessitating action. In many if not most cases, these species are not the focus of ongoing conservation efforts. With a requirement for the development of recovery plans and funding mechanisms for recovery action, the ESA would provide distinct benefits for these species.

Like the listing process itself, our methodology includes some degree of subjectivity in identifying species that need consideration for ESA protections. Regan et al. (2013) proposed quantitative decision rules as a means of making listing decisions more objective and repeatable, but these and similar methods have gained little to no traction because they are divorced from the statutory requirements of the ESA and lack practicability in the real world. The ESA requires consideration of five threat factors that are not discussed by the authors, and in our view astutely focus analysis on anthropogenic actions that are often unpredictable but can quickly drive species to extinction, rather than quantified measures of extinction probability based on species’ demographics, which are in many if not most cases unavailable. The ESA requires consideration of the best available information, which by necessity includes both qualitative and quantitative data.

## Conclusions

In this study, we seek to provide an efficient methodology that aligns with the statute and facilitates identification of species where there is sufficient information about threats such that a review for listing is warranted. This is an important first step that bridges the gap between the more than 10,000 species identified by NatureServe as imperiled and the initial step in the listing process. We believe our identification of 2,204 species is conservative with the true number of species needing not just consideration for protection under the ESA, but listing and full protection being much higher.

Indeed, based on an extrapolation of known rates of imperilment of well-studied groups and estimated numbers of species overall, Wilcove & Master (2005) estimated there could be anywhere between 14,000 and 35,000 endangered species in the US. The substantial gap between this estimate and our figure of 2,204 species reflects the limited knowledge available for most species, including substantial numbers that have yet to be described. Notably, a large percentage (approaching 80 percent) of species recognized as critically imperiled or imperiled by NatureServe likely lack sufficient threat information to be considered for listing, which is not to say they are not facing threats. While NatureServe in some cases rates species as imperiled solely based on rarity, Wilcove et al. (1998) found that just 52 of 1,880 critically imperiled or imperiled species lacked anthropogenic threats, suggesting most imperiled species likely need protection. Clearly, a massive increase in funding for research into the status of US species is needed if we are to truly protect our natural heritage.

In the absence of such funding, the 2,204 species identified in this study represent a pared down list of species needing immediate consideration for listing as endangered or threatened species. Our methodology is an effective means for identifying imperiled species that need consideration for the regulatory protections provided by the ESA.

##  Supplemental Information

10.7717/peerj.20692/supp-1 Supplemental Information 1 Results to date of Southeast species petition

10.7717/peerj.20692/supp-2 Supplemental Information 2 NatureServe accounts for 2,206 species identified as needing consideration for listing under the Endangered Species Act

## Additional Information and Declarations

### Data Availability

The following information was supplied regarding data availability: The data is available in the Supplemental Files.
